# β-Adrenergic regulation of cardiac type 2A protein phosphatase through phosphorylation of regulatory subunit B56δ at S573

**DOI:** 10.1016/j.yjmcc.2017.12.016

**Published:** 2018-02

**Authors:** Antonella Ranieri, Elizabeth Kemp, Joseph R. Burgoyne, Metin Avkiran

**Affiliations:** School of Cardiovascular Medicine and Sciences, King's College London British Heart Foundation Centre of Research Excellence, The Rayne Institute, St Thomas' Hospital, London, United Kingdom

**Keywords:** ARVM, adult rat ventricular myocytes, CaMKII, calcium/calmodulin-dependent kinase II, cMyBP-C, cardiac myosin binding protein-C, cTnI, cardiac troponin I, I-1, inhibitor of protein phosphatase-1, ISO, isoprenaline, KO, knock out, MOI, multiplicity of infection, BNZ, N^6^-benzoyl-cAMP, PLB, phospholamban, PKA, protein kinase A, Ser, S, serine, Thr, T, threonine, PP1, type 1 phosphatase, PP2A, type 2A protein phosphatase, WT, wild type, βAR, β-Adrenergic receptor, β-Adrenergic signaling, B56δ, Cardiomyocytes, Phosphatase, Phosphorylation, Type 2A protein phosphatase (PP2A), Protein kinase A (PKA)

## Abstract

**Background:**

Type 2A protein phosphatase (PP2A) enzymes are serine/threonine phosphatases which comprise a scaffold A subunit, a regulatory B subunit and a catalytic C subunit, and have been implicated in the dephosphorylation of multiple cardiac phosphoproteins. B subunits determine subcellular targeting, substrate specificity and catalytic activity, and can themselves be regulated by post-translational modifications. We explored potential β-adrenergic regulation of PP2A in cardiomyocytes through phosphorylation of the regulatory B subunit isoform B56δ.

**Methods and results:**

Phosphate affinity SDS-PAGE and immunoblot analysis revealed increased phosphorylation of B56δ in adult rat ventricular myocytes (ARVM) exposed to the β-adrenergic receptor (βAR) agonist isoprenaline (ISO). Phosphorylation of B56δ occurred at S573, primarily through stimulation of the β_1_AR subtype, and was dependent on PKA activity. The functional role of the phosphorylation was explored in ARVM transduced with adenoviruses expressing wild type (WT) or non-phosphorylatable (S573A) B56δ, fused to GFP at the N-terminus. C subunit expression was increased in ARVM expressing GFP-B56δ-WT or GFP-B56δ-S573A, both of which co-immunoprecipitated with endogenous C and A subunits. PP2A activity in cell lysates was increased in response to ISO in ARVM expressing GFP-B56δ-WT but not GFP-B56δ-S573A. Immunoblot analysis of the phosphoproteome in ARVM expressing GFP-B56δ-WT or GFP-B56δ-S573A with antibodies detecting (i) phospho-serine/threonine residues in distinct kinase substrate motifs or (ii) specific phosphorylated residues of functional importance in selected proteins revealed a comparable phosphorylation profile in the absence or presence of ISO stimulation.

**Conclusions:**

In cardiomyocytes, βAR stimulation induces PKA-mediated phosphorylation of the PP2A regulatory subunit isoform B56δ at S573, which increases associated PP2A catalytic activity. This is likely to regulate the phosphorylation status of specific B56δ-PP2A substrates, which remain to be identified.

## Highlights

• PP2A subunit B56δ is phosphorylated on β-adrenergic stimulation of cardiomyocytes.
• Phosphorylation occurs at Ser573 and increases B56δ-PP2A catalytic activity.
• Response is mediated by the β_1_-adrenoceptor subtype and protein kinase A.
• Phosphorylated B56δ abundance is increased in pathological cardiac hypertrophy.

## 1 Introduction

Type 2A protein phosphatase (PP2A) holoenzymes are present in most cell types, including cardiac myocytes, where they dephosphorylate phospho-serine (Ser, S) and phospho-threonine (Thr, T) residues in proteins. PP2A holoenzymes comprise a 65-kDa scaffold (PP2A_A_ or A) subunit, a 36-kDa catalytic (PP2A_C_ or C) subunit and a regulatory (PP2A_B_ or B) subunit of variable molecular weight that determines subcellular targeting, substrate specificity and catalytic activity [1], [2], [3], [4]. The importance of B subunits and regulated catalytic activity in the heart is highlighted by the dilated cardiomyopathy phenotype of mice expressing a mutant A subunit that binds the C but not the B subunit [5], and by the impaired cardiac function of mice with cardiomyocyte-specific overexpression of the C subunit [6].

B subunits are classified into three families, PR55/B, PR61/B′ and PR72/B″. The largest of these is the PR61/B′ family, which is commonly referred to as the B56 family and comprises α, β, γ, δ and ε isoforms [2]. Conserved sequences residing in the central domain of B56 isoforms enable association with the AC dimer, whilst unique sequences at the N- and C-terminals determine isoform-specific functions [7], [8]. Regarding the role of individual isoforms in the heart in vivo, studies in mice with global deletion [9] or cardiac-specific overexpression [10] of B56α indicate that this B subunit regulates contractility, potentially through phospho-regulation of proteins involved in excitation-contraction (EC) coupling. Studies in mice with global deletion of B56γ indicate that this isoform regulates cardiac development [11].

The focus of this study is B56δ. The gene encoding B56δ in humans (PPP2R5D) is localized in chromosome region 6p21.1 and gives rise to three splice variants that differ at the N-terminus [12], [13], [14]. With 602 amino acids and a predicted molecular weight of 70-kDa, δ1 is the largest splice variant. Relative to this splice variant, δ2 and δ3 lack amino acids 84-115 and 1-115, respectively. Studies in vitro have shown that the activity of B56δ-PP2A can be increased by protein kinase A (PKA), through phosphorylation of B56δ at S60, S75, S88 and S573 [15], [16], [17]. In non-cardiac cells, B56δ phosphorylation at S573 is necessary for increased B56δ-PP2A activity and is induced by G protein-coupled receptors that signal via the Gs-adenylate cyclase-cAMP-PKA pathway such as dopamine D1 and luteinizing hormone receptors [16], [18].

Little information is available regarding B56δ expression, regulation and function in the heart. Mice lacking B56δ were the first mice with targeted disruption of a B56 subunit to be studied; however, their cardiac phenotype was not studied [19]. More recently, DeGrande et al. [20] have shown that B56δ protein is expressed in mammalian heart chambers, and that its levels are increased in dog hearts with ischemic or non-ischemic heart failure.

In view of the importance of the Gs-adenylate cyclase-cAMP-PKA pathway in mediating cardiomyocyte responses to β-adrenergic receptor (βAR) stimulation [21], [22], we have explored potential β-AR-mediated regulation and role of B56δ phosphorylation in adult rat ventricular myocytes (ARVM). We have found that (i) B56δ is phosphorylated at S573 following the acute stimulation of βARs, (ii) this response occurs primarily downstream of the β_1_AR and is mediated by PKA, and (iii) B56δ phosphorylation at S573 is necessary for βAR-mediated stimulation of PP2A activity.

## 2 Materials and methods

### 2.1 Materials

cTnI, pS23/24 cTnI, PP2A_C_, PLB and phospho-S/T kinase substrate antibodies were from Cell Signaling Technology (2002, 4002, 2038, 8495 and 9920, respectively). GAPDH, H2B and B56γ antibodies were from Abcam (Ab9482, Ab1790-100 and Ab94633, respectively). B56α and B56δ antibodies were from BD Biosciences (610615) and Bethyl (A301-100A), respectively. PP2A_A_ and B55α antibodies were from Santa Cruz (sc-74580 and sc-81606, respectively). GFP and α-Actinin antibodies were from Roche (11814460001) and Sigma (A7732), respectively. pS16 PLB and pS282 cMyBP-C antibodies were from Badrilla (A010-12AP) and Enzo Life Sciences (ALX-215-057-R050), respectively. The cMyBP-C antibody was a kind gift from Professor Mathias Gautel [23]. pS273 and pS302 cMyBP-C antibodies were a kind gift from Dr. Jeffrey Robbins [24], [25]. The pS573 B56δ antibody was a kind gift from Professor Angus Nairn [16]. Cy3- and Cy5-conjugated secondary antibodies were from Jackson ImmunoResearch (115165146 and 111175144, respectively). Isoprenaline (ISO), propranolol (PRO), CGP-20712A (CGP) and ICI 118,551 (ICI) were from Sigma (I5627, P0084, C231 and I127, respectively). H89, myristoylated PKA inhibitor 14-22 amide (PKI), N^6^-benzoyl cAMP (BNZ), and okadaic acid were from Calbiochem (371962, 476485, 116802 and 495609, respectively). The pEGFP-C1 vector encoding *PPP2R5D* and cardiac tissue from littermate B56δ knock out (KO) and WT mice were kind gifts from Professor Veerle Janssens [19]. Adult male Wistar rats (300–324 g) were from Harlan Laboratories (UK).

### 2.2 Construction of adenoviral vectors

To replace S573 with alanine, a single point mutation was introduced into human *PPP2R5D* in a pEGFP-C1 vector using the QuikChange II Site-Directed Mutagenesis Kit (Stratagene). The adenoviral vectors expressing wild type (WT) B56δ (AdV.GFP-B56δ-WT) and mutated (S573A) B56δ (AdV.GFP-B56δ-S573A) were constructed using the AdEasy system [26]. In brief, GFP-B56δ cDNA was subcloned into pShuttle-CMV (Stratagene) and homologous recombination of this with pAdEasy-1 (Stratagene) was performed in bacterial cells. Adenoviruses were amplified in HEK293 cells and purified using a cesium chloride density gradient in combination with ultracentrifugation. The infectious titer of the purified adenoviruses was determined in tissue culture infectivity dose 50 assays [27].

### 2.3 Isolation, culture and adenoviral transduction of ARVM

ARVM were isolated from the hearts of adult male Wistar rats by collagenase-based enzymatic digestion, as previously described [28], [29]. Isolated cells were resuspended in Hank's M199 medium supplemented with 2 mM l-creatine, 5 mM carnitine, 5 mM taurine and 100 IU/ml penicillin/streptomycin, and were cultured in plastic 6-well plates pre-coated with laminin. Cells were maintained in a humidified incubator (5% CO_2_, 37 °C) for 2 h after which, the medium was replaced with fresh medium and cells were incubated overnight. Where indicated, cells were transduced with adenoviruses 2 h post-plating. AdV.GFP was used at MOI 30. AdV.GFP-B56δ-WT and AdV.GFP-B56δ-S573A were both used at MOI 100.

### 2.4 Pharmacological treatment of ARVM

Unless otherwise stated, ARVM were incubated with vehicle or 10 nM ISO for 10 min. PRO (100 nM), CGP (100 nM), ICI (100 nM) or vehicle was added to the culture medium 10 min before ISO stimulation. H89 (10 μM), PKI (10 μM) or vehicle was added 30 min before ISO stimulation. Cells were exposed to BNZ (500 μM) or vehicle for 30 min, and to OA (0.1 or 1 μM) or vehicle for 60 min. Experiments were performed at 37 °C.

### 2.5 Subcellular fractionation of ARVM

The subcellular fractionation method was adapted from methods described in previous publications [29], [30]. In brief, cells were harvested in ice-cold lysis buffer containing: 50 mM Tris (pH 7.5), 5 mM EGTA, 2 mM EDTA, 100 mM NaF, 1% (v/v) Triton-X100 and complete mini protease inhibitor (Roche). Cell lysates were incubated on ice for 5 min after which, they were centrifuged at 14,000*g* for 30 min at 4 °C. Proteins in the soluble fraction (supernatant) were denatured in 3X Laemmli sample buffer. Proteins in the insoluble fraction (pellet) were resuspended in 1X Laemmli sample buffer.

### 2.6 SDS-PAGE and immunoblot analysis

Heat-denatured protein samples were resolved on Tris-glycine SDS-PAGE gels and transferred to PVDF membranes. Membranes were blocked in Tris-buffered saline with 0.1% Tween-20 (TBST) and 5% (w/v) non-fat milk. Incubation with primary antibodies was performed overnight at 4 °C and incubation with secondary antibodies was performed for 2 h at room temperature. Protein bands were visualized on chemiluminescence film following incubation of the membranes with ECL Western Blotting Detection Reagents (GE Healthcare). Signal intensity was quantified on a calibrated GS-800 densitometer (Bio-Rad). Phosphate affinity (PhosTag) SDS-PAGE was performed on Tris-glycine gels containing 50 μM acrylamide-pendent PhosTag™ and 100 μM MnCl_2_. Prior to transfer of proteins to PVDF membranes, gels were incubated 15 min in transfer buffer containing 1 mM EDTA then 15 min in transfer buffer alone.

### 2.7 Immunolabeling and imaging of ARVM

ARVM were prepared for imaging on a confocal florescence microscope as previously described [31]. Unless otherwise stated, incubations were performed at room temperature. In brief, cells were incubated with (i) 4% paraformaldehyde for 10 min, (ii) 0.2% (v/v) Triton-X100 for 5 min, and (iii) 5% normal goat serum for 20 min. Incubation with primary antibodies (or control IgG) was performed overnight at 4 °C. Incubation with Cy3- and Cy5-conjugated secondary antibodies was performed for 3 h. Cells were overlaid with coverslips in a mounting media containing the anti-fading reagent n-propyl gallate [32]. Immunolabeled cells were imaged on a Leica TCS SP5 confocal microscope equipped with a UV-diode, an argon laser and a helium-neon laser, as previously described [29], [31].

### 2.8 Immunoprecipitation of GFP, GFP-B56δ-WT and GFP-B56δ-S573A

GFP, GFP-B56δ-WT and GFP-B56δ-S573A were immunoprecipitated from ARVM lysates using a GFP antibody. Cells were harvested in ice-cold lysis buffer containing: 50 mM Tris (pH 8), 138 mM NaCl, 27 mM KCl, 1% (v/v) Triton-X100 and complete mini protease inhibitor. The cleared lysate containing 500 μg of protein was incubated with anti-GFP (4 μg) over-night at 4 °C. Immunocomplexes were collected using Protein G beads. After three washes in lysis buffer, these were resuspended in Laemmli buffer.

### 2.9 Measurement of protein phosphatase activity

Protein phosphatase activity was determined using the SensoLyte *p*NPP Protein Phosphatase Assay kit (AnaSpec), as recommended by the manufacturer. Cells expressing heterologous proteins were washed in phosphate-buffered saline and harvested in 1X lysis buffer with added 1% (v/v) Triton-X100 and protease inhibitors. Master mixes of whole cell lysates in assay buffer (40 mM Tris (pH 8.4), 34 mM MgCl_2_, 4 mM EDTA and 4 mM DTT) were prepared on ice. Incubation with 5 nM okadaic acid or DMSO was performed for 10 min. Assays were performed in triplicates, in wells of 96-well plates. Individual wells contained 10 μg of protein in 50 μl of master mix and 50 μl of *p*NPP solution. Absorbance at 405 nm was determined in a plate reader pre-heated to 30 °C. Measurements were recorded at 5 min intervals, for a total of 30 min. The amount of *p*NP produced was determined using the equation c = A ÷ (ɛ × l), where c is the concentration of *p*NP, A is the optical absorbance of *p*NP at 405 nm, ɛ is the molar extinction coefficient of *p*NP (18,000 M^− 1^ cm^− 1^) and l is the path length.

### 2.10 Mouse pressure overload hypertrophy model

Transverse aortic constriction (TAC) surgery was performed in adult mice by Professor Brian Cooley (Animal Surgery Core Laboratory, McAllister Heart Institute, UNC School of Medicine). Male C57Bl/6 mice at 9 weeks of age were anaesthetised by intraperitoneal injection of 100 mg/kg ketamine and 15 mg/ml xylazine. Constriction of the aortic arch was achieved by ligation of the aorta with 6-0 silk, using a 27.5-gauge spacer. Sham animals were subjected to the same surgical procedure, with no constrictive suture. Hearts were harvested 7 days after the surgical procedure and frozen in liquid N_2_ for subsequent analysis.

### 2.11 Data analysis

Quantitative data are presented as mean ± SEM. Differences between groups were analysed by unpaired *t*-test, or One-Way ANOVA followed by Tukey's multiple comparisons test. *P* < 0.05 was considered significant. In figure legends, *n* indicates the number of independent experiments.

## 3 Results

### 3.1 The effect of ISO on phosphorylation of B56δ in ARVM

**Fig. 1:**
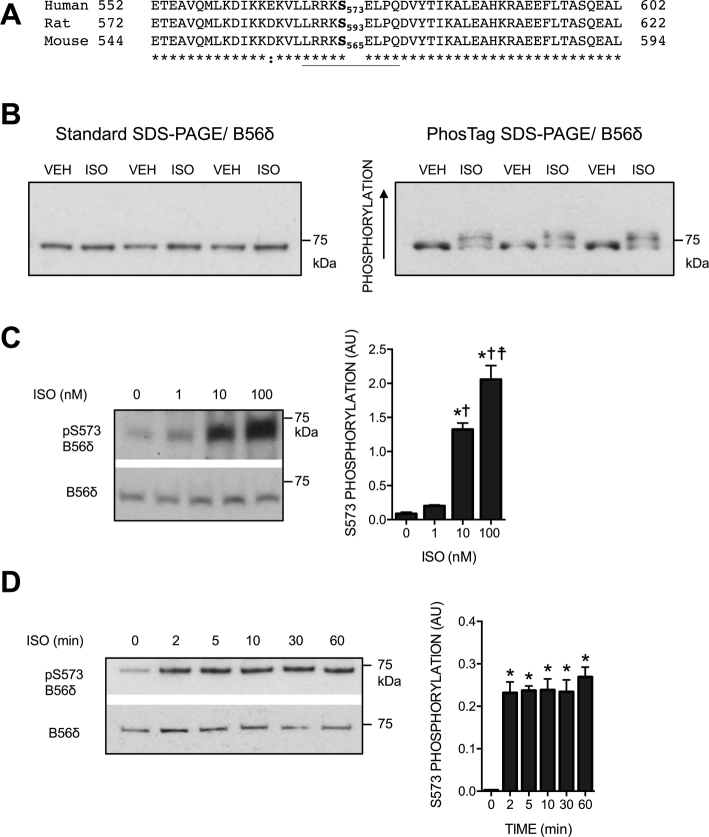
ISO-induced B56δ phosphorylation in ARVM. A. Amino acids at the C-terminus of human (UniProt entry Q14738), rat (UniProt entry Q499R1) and mouse (UniProt entry Q91V89) B56δ isoforms. Asterisks denote identical amino acids; phosphorylatable serine residues (S573 in the human, S593 in the rat and S565 in the mouse isoform) are in bold. The polyclonal B56δ antibody is raised against a sequence of amino acids between amino acids 552 and 602 of the human isoform. The phospho-S573 B56δ antibody is raised against a phospho-peptide comprising the underlined residues. B. Lysates of ARVM exposed to vehicle (VEH) or isoprenaline (ISO) were subjected to phosphate affinity (PhosTag) SDS-PAGE and standard SDS-PAGE. Immunoblot analysis was performed with a B56δ antibody. C. ARVM were exposed to 1 nM, 10 nM or 100 nM ISO. Figure shows representative immunoblots and quantitative data (mean ± SEM) for B56δ phosphorylation at S573. *P < 0.05 vs 0 nM ISO, ^✝^P < 0.05 vs 1 nM ISO, ^☨^P < 0.05 vs 10 nM ISO (*n* = 3). D. ARVM were exposed to 10 nM ISO for 2, 5, 10, 30 or 60 min. Figure shows representative immunoblots and quantitative data (mean ± SEM) for B56δ phosphorylation at S573. *P < 0.05 vs 0 min (*n* = 3).

To explore the effect of βAR stimulation on B56δ phosphorylation in cardiomyocytes, cell lysates from unstimulated and ISO-stimulated ARVM were resolved by standard- and PhosTag SDS-PAGE. Immunoblot analysis was performed with a polyclonal antibody raised against a peptide sequence at the C-terminus (aa 552-602) of human B56δ, which is conserved in the rat and mouse protein (Fig. 1A). When proteins were resolved by standard SDS-PAGE, a single protein migrating at ~ 70-kDa was detected in all samples, indicating that ARVM express only the δ1 splice variant (Fig. 1B, left panel). When PhosTag SDS-PAGE was used, an additional B56δ band that migrated more slowly was detected in ISO-stimulated cells, indicating increased B56δ phosphorylation (Fig. 1B, right panel). Further studies of the specific site of phosphorylation were guided by the evidence that in primary non-cardiac cells phosphorylation occurs at S573 [16]. As indicated in Fig. 1A, S573 in human B56δ corresponds to S593 in the rat protein and S565 in the mouse protein. To determine phosphorylation of this conserved residue in ARVM, we used a previously described phospho-specific antibody that recognizes human B56δ phosphorylated at S573 (the target phospho-motif is underlined in Fig. 1A) [16]. ISO induced a concentration-dependent increase in S573 phosphorylation that was significant at concentrations ≥ 10 nM (Fig. 1C). With 10 nM ISO, maximum phosphorylation was achieved within 2 min of stimulation and the response was sustained for at least 60 min (Fig. 1D). These data indicate that in ARVM stimulation of βARs induces rapid and robust phosphorylation of B56δ at S573. Confirming the specificity of the total and phospho-S573 B56δ antibodies, the protein detected by these antibodies at ~ 70-kDa in ARVM and WT mouse heart was not detected in B56δ KO^19^ mouse heart (Supplementary Fig. 1).

### 3.2 The role of βAR subtypes and PKA in ISO-induced phosphorylation of B56δ

**Fig. 2:**
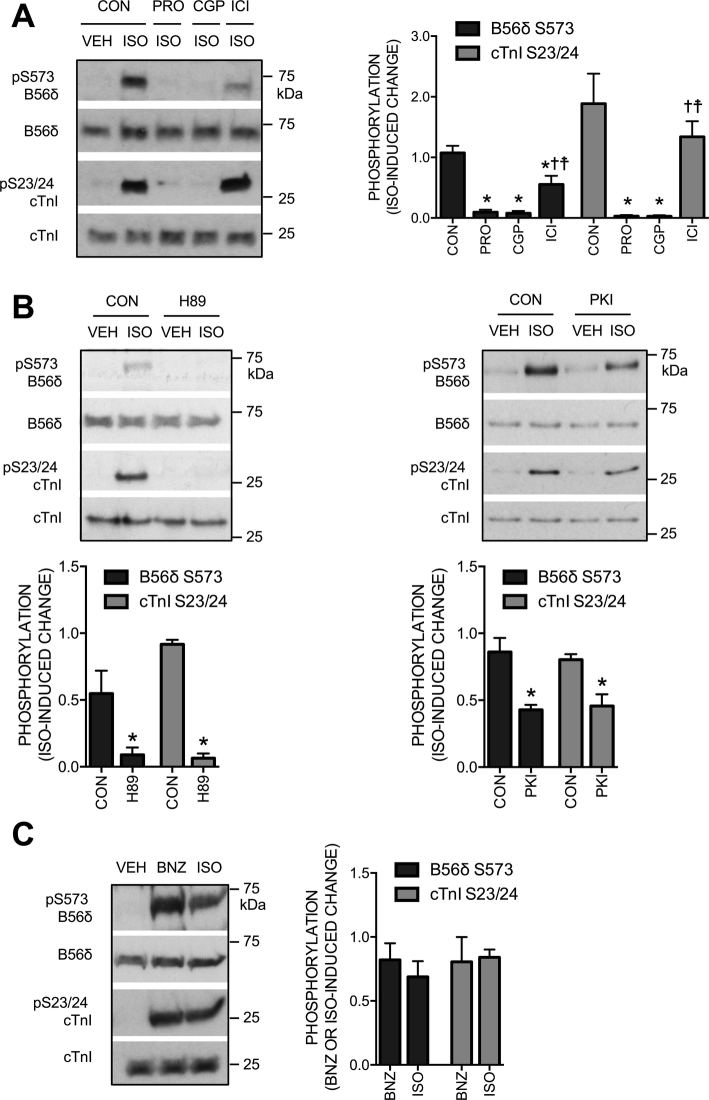
The role of βAR subtypes and PKA in ISO-induced B56δ phosphorylation. A. ARVM were exposed to vehicle (VEH) or isoprenaline (ISO), following incubation with a βAR antagonist or vehicle control (CON). Representative immunoblots and quantitative data (mean ± SEM) show the effect of the non-selective βAR antagonist propranolol (PRO, 100 nM), the β_1_AR-selective antagonist CGP 20712A (CGP, 100 nM) and the β_2_AR-selective antagonist ICI 118,551 (ICI, 100 nM) on the ISO-induced change in B56δ phosphorylation at S573 (black bars) and cTnI phosphorylation at S23/24 (grey bars). *P < 0.05 vs CON, ^✝^P < 0.05 vs PRO, ^☨^P < 0.05 vs CGP (*n* = 7). B. ARVM were exposed to VEH or ISO, following incubation with a PKA inhibitor or CON. Representative immunoblots and quantitative data (mean ± SEM) show the effect of the PKA inhibitors H89 (10 μM) and PKI (10 μM) on the ISO-induced change in B56δ phosphorylation at S573 (black bars) and cTnI phosphorylation at S23/24 (grey bars). *P < 0.05 vs CON (*n* = 4). C. Representative immunoblots and quantitative data (mean ± SEM) show the effect of the PKA-selective analogue of cAMP N^6^-benzoyl cAMP (BNZ, 500 μM) and ISO on B56δ phosphorylation at S573 (black bars) and cTnI phosphorylation at S23/24 (grey bars) (*n* = 3).

We next investigated the signaling mechanisms that underlie ISO-induced B56δ phosphorylation at S573. The response was blocked by propranolol, a non-selective βAR antagonist, confirming a βAR-mediated mechanism (Fig. 2A). To determine the contributions of individual βAR subtypes, we used CGP 20712A (CGP) and ICI 118,551 (ICI) to selectively block β_1_ or β_2_ARs, respectively. ISO-induced B56δ phosphorylation at S573 was abolished by CGP and partially attenuated by ICI (Fig. 2A). Reflecting the established βAR subtype selectivity of the antagonists [33], [34], phosphorylation of cardiac troponin I (cTnI) at S23/24, the sites phosphorylated by PKA downstream of β_1_ARs [35], was abolished by CGP but was not affected by ICI (Fig. 2A). Guided by the evidence that PKA phosphorylates B56δ at S573 in non-cardiac cells [16], we determined the role of PKA in cardiac B56δ phosphorylation at this site. To inhibit PKA in ARVM, we incubated cells with the ATP binding-site competitor H89 or the inhibitory peptide PKI before stimulation with ISO. The ISO-induced increase in B56δ S573 phosphorylation was abolished by H89 (Fig. 2B, left panel) and significantly attenuated by PKI (Fig. 2B, right panel). Importantly, H89 and PKI inhibited the ISO-induced increase in cTnI phosphorylation to a similar extent, indicating that the observed reductions in B56δ phosphorylation reflected the extent of PKA inhibition achieved by the inhibitors. In complementary studies, we used the PKA-selective analogue of cAMP, N^6^-benzoyl-cAMP (BNZ) [36], to determine the effect of direct PKA activation on B56δ S573 phosphorylation. The increase in S573 phosphorylation induced by BNZ was comparable to that induced by 10 nM ISO (Fig. 2C). Together, these data suggest that activation of PKA is both necessary and sufficient for B56δ phosphorylation at S573 in ARVM.

### 3.3 Subcellular distribution of B56δ in ARVM

**Fig. 3:**
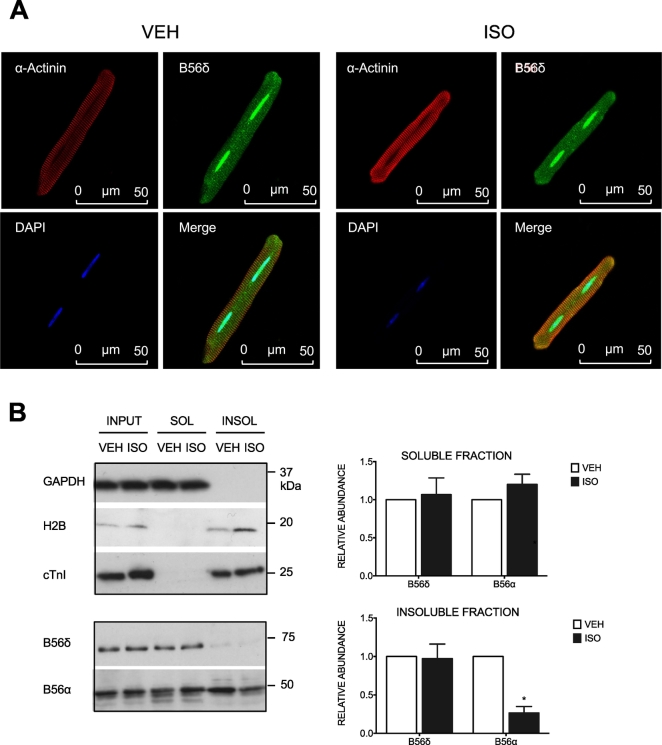
Subcellular distribution of B56δ in ARVM. In A and B, ARVM were exposed to vehicle (VEH) or isoprenaline (ISO). A. Fixed and permeabilized cells were incubated with α-actinin and B56δ primary antibodies. These were detected with Cy3-anti-mouse and Cy5-anti-rabbit secondary antibodies, respectively. Nuclei were stained with DAPI. Representative images show Cy3-labeled α-actinin (red), Cy5-labeled B56δ (green) and DAPI-stained nuclei (blue) in separate channels. Merged images are also shown. B. Cells lysed in buffer containing 1% Triton-X100 were subjected to subcellular fractionation. Immunoblots show detection of GAPDH (cytosolic protein), H2B (nuclear protein), cTnI (myofilament protein), B56δ and B56α in soluble (SOL) and insoluble (INSOL) subcellular fractions. Quantitative data (mean ± SEM) show B56δ and B56α abundance in soluble and insoluble fractions. In each experiment, protein abundance in the ISO group was expressed relative to that in the corresponding VEH group. *P < 0.05 vs VEH (*n* = 6). (For interpretation of the references to colour in this figure legend, the reader is referred to the web version of this article.)

We have shown previously that ISO stimulation in ARVM induces a change in the subcellular distribution of a different B56 isoform, B56α [29]. To determine if ISO alters subcellular distribution of B56δ, we used fluorescence microscopy to visualise the immunolabeled protein in intact myocytes. B56δ was found throughout the cytosol and was present in nuclei, in both unstimulated and ISO-stimulated cells (Fig. 3A). The specificity of the rabbit B56δ antibody was confirmed by incubating cells with an equivalent concentration of non-immune rabbit IgG (Supplementary Fig. 2). In complementary experiments, we determined the distribution of B56δ in soluble and insoluble compartments of fractionated cells (Fig. 3B). The purity of the soluble and insoluble fractions was determined by immunoblot analysis of proteins with known subcellular compartmentalization, with GAPDH (a cytosolic enzyme) selected as the marker for the soluble fraction and histone 2B (H2B, a nuclear, DNA-bound protein) and cTnI (a myofilament protein) selected as markers for the insoluble fraction. Although shown to be present in the nuclei of intact cells, B56δ was detected primarily in the soluble subcellular fraction, suggesting labile nuclear interactions. Furthermore, the abundance of B56δ in the soluble and insoluble fraction was unchanged in response to ISO. In contrast, and consistent with our previous work [29], B56α abundance in the insoluble fraction was reduced following ISO stimulation. Together, these data suggest that increased phosphorylation of B56δ at S573 does not alter its subcellular distribution in ARVM.

### 3.4 Adenoviral expression of GFP-B56δ-WT and GFP-B56δ-S573A in ARVM

**Fig. 4:**
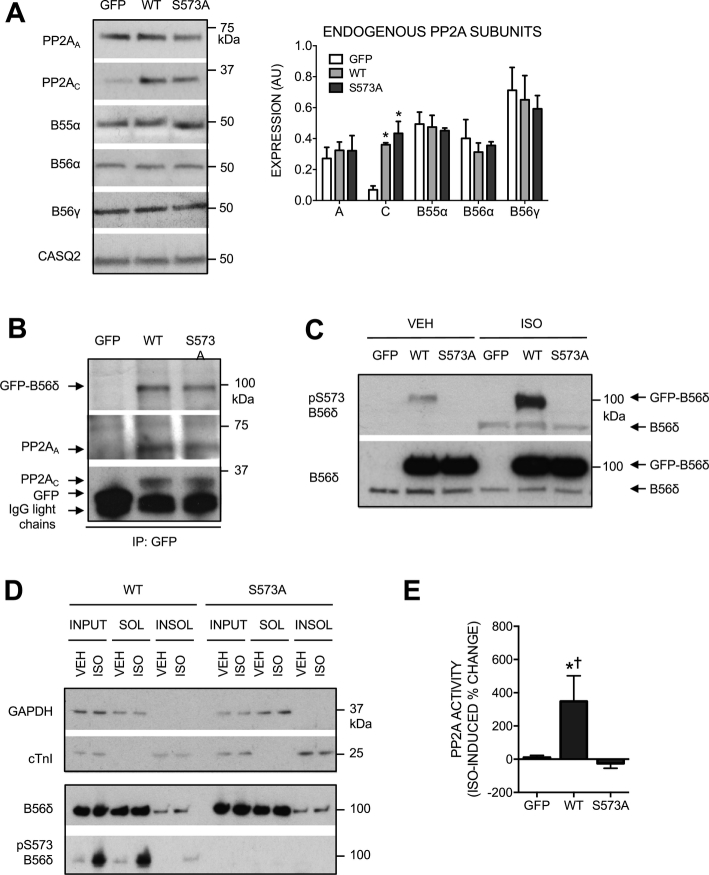
Characterization of adenoviral vectors. ARVM transduced with AdV.GFP, AdV.GFP-B56δ-WT or AdV.GFP-B56δ-S573A were maintained in culture for 18 h. In C, D and E, cells were exposed to vehicle (VEH) or isoprenaline (ISO). A. Expression of PP2A_A_, PP2A_C_, B55α, B56α and B56γ in transduced cells. Quantitative data (mean ± SEM) show endogenous PP2A subunit expression normalized to the expression of calsequestrin. *P < 0.05 vs GFP (*n* = 3). B. GFP, GFP-B56δ-WT and GFP-B56δ-S573A were immunoprecipitated from lysates of transduced cells with a GFP antibody. GFP-B56δ-WT, GFP-B56δ-S573A, GFP, PP2A_A_ and PP2A_C_ were detected by immunoblot analysis. C. Phosphorylation of S573 in heterologously expressed B56δ. ISO-induced phosphorylation of S573 in heterologous (GFP-B56δ) and endogenous B56δ is indicated. D. Cells lysed in buffer containing 1% Triton-X100 were subjected to subcellular fractionation. Immunoblots show detection of GAPDH, cTnI, GFP-B56δ-WT and GFP-B56δ-S573A in soluble (SOL) and insoluble (INSOL) subcellular fractions. The distribution of GFP-B56δ-WT phosphorylated at S573 is also shown. E. Protein phosphatase activity was measured in whole cell lysates using *p*NPP as substrate. The specific activity of PP2A was determined by using 5 nM okadaic acid. Quantitative data (mean ± SEM) show the ISO-induced change in PP2A activity. In each experiment, the change in PP2A activity was expressed as a percentage of the basal activity. *P < 0.05 vs GFP, ^✝^P < 0.05 vs SA (*n* = 6).

To study the functional importance of B56δ phosphorylation at S573, we constructed adenoviral vectors for heterologous expression of GFP-tagged WT or non-phosphorylatable (S573A) B56δ (AdV.GFP-B56δ-WT and AdV.GFP-B56δ-S573A, respectively) in ARVM. Control cells were transduced with an adenovirus that expresses GFP alone (AdV.GFP). Comparable GFP protein expression was achieved following 18 h incubation with AdV.GFP at multiplicity of infection (MOI) 30 and AdV. GFP-B56δ-WT or AdV.GFP-B56δ-S573A at MOI 100 (Supplementary Fig. 3A). Immunoblot analysis of the same samples with the B56δ antibody confirmed comparable expression of GFP-B56δ-WT and GFP-B56δ-S573A, and showed that, relative to cells expressing GFP alone, expression of endogenous B56δ was unchanged (Supplementary Fig. 3B). To further characterize the adenoviral vectors, we determined protein levels of endogenous PP2A subunits in transduced cells. Expression of GFP-B56δ-WT and GFP-B56δ-S573A was associated with increased expression of C subunits, but no change in the expression of A subunits. Expression of other B subunit isoforms (B55α, B56α and B56γ) was also unchanged (Fig. 4A). To determine whether GFP-B56δ-WT and GFP-B56δ-S573A were incorporated into PP2A holoenzymes, protein complexes were immunoprecipitated from lysates of transduced cells using a GFP antibody and the resulting samples were subjected to SDS-PAGE and immunoblot analysis. Both A and C subunits were detected (in comparable abundance) in samples from cells expressing GFP-B56δ-WT and GFP-B56δ-S573A, suggesting that the GFP-tagged B56δ variants were indeed incorporated into PP2A holoenzymes within ARVM (Fig. 4B). To characterize the phosphorylation status of GFP-B56δ-WT and GFP-B56δ-S573A in ARVM, immunoblot analysis of unstimulated and ISO-stimulated cells was performed with the phospho-S573 B56δ antibody. Basal and ISO-induced phosphorylation of S573 was detected in GFP-B56δ-WT but not GFP-B56δ-S573A, confirming loss of the phosphorylatable residue in the latter protein (Fig. 4C). ISO-induced phosphorylation of S573 in endogenous B56δ was detected simultaneously and was comparable in all groups (Fig. 4C).

In the HEK293 cell line, PKA activation has been reported to induce B56δ phosphorylation not just at S573 but also at S88, albeit to a much smaller extent [16]. In human B56δ, both S573 and S88 are preceded by arginine (R) residues at the − 2 and − 3 positions, forming optimal PKA substrate motifs (Supplementary Fig. 4A). To determine if S573 is the only site that is phosphorylated in heterologously expressed human B56δ in ARVM, GFP-B56δ-WT or GFP-B56δ-S573A immunoprecipitated from lysates of unstimulated and ISO-stimulated cells was subjected to immunoblot analysis with a phospho-specific antibody that detects phospho-S/T (S/T) residues in the RRXS/T motif. This antibody detected basal S/T phosphorylation in GFP-B56δ-WT and the signal was markedly increased following ISO stimulation (Supplementary Fig. 4B). In contrast, the antibody did not detect GFP-B56δ-S573A in the absence or presence of ISO stimulation (Supplementary Fig. 4B), indicating that in ARVM S573 is the only B56δ site that is phosphorylated.

To explore whether ablation of the S573 phosphorylation site impacts on B56δ subcellular localization, we determined the distribution of GFP-B56δ-WT and GFP-B56δ-S573A in soluble and insoluble compartments of fractionated cells. As in earlier experiments (Fig. 3B), the purity of the soluble and insoluble fractions was confirmed by immunoblot analysis of GAPDH and cTnI, respectively (Fig. 4D). Like endogenous B56δ (Fig. 3B), GFP-B56δ-WT was detected primarily in the soluble fraction and did not show translocation upon ISO-induced phosphorylation at S573 (Fig. 4D). The subcellular distribution of GFP-B56δ-S573A was identical to that of GFP-B56δ-WT, in the absence and presence of ISO stimulation (Fig. 4D). These findings indicate that ablation of the S573 phosphorylation site does not impact on the subcellular localization heterologously expressed GFP-B56δ, which behaves in an analagous manner to endogenous B56δ.

Studies in vitro and in non-cardiac cells have shown that B56δ phosphorylation at S573 increases the activity of its associated C subunit [16]. To determine the impact of S573 phosphorylation in cardiomyocytes, we determined PP2A catalytic activity in lysates of unstimulated and ISO-stimulated ARVM expressing GFP, GFP-B56δ-WT or GFP-B56δ-S573A. PP2A activity was defined as the activity inhibited by 5 nM okadaic acid (OA), a concentration that in vitro is sufficient to inhibit the activity of PP2A but not PP1 [37]. PP2A activity was increased by ISO only in cells expressing GFP-B56δ-WT (Fig. 4E), indicating that B56δ phosphorylation at S573 is necessary for increasing associated C subunit activity in response to β-adrenergic stimulation.

### 3.5 The role of S573 phosphorylation in regulation of cardiac protein phosphorylation

**Fig. 5:**
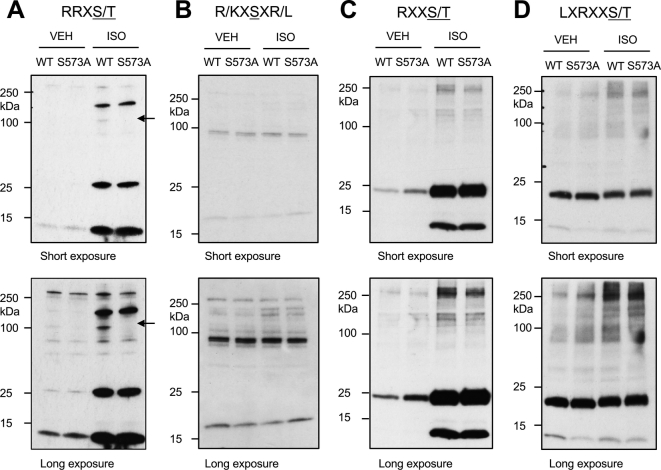
Protein phosphorylation in transduced ARVM. ARVM transduced with AdV.GFP-B56δ-WT or AdV.GFP-B56δ-S573A were maintained in culture for 18 h and exposed to vehicle (VEH) or isoprenaline (ISO). Immunoblot analysis of the ARVM phosphoproteome was performed with phospho-specific antibodies detecting phospho-S/T residues in the A. RRXS/T motif; B. R/KXSXR/L motif; C. RXXS/T motif; and D. LXRXXS/T motif. In each panel, short and long exposures of the film to the ECL signal are shown. In A, arrows indicate phosphorylation of B56δ in GFP-B56δ-WT. L = leucine, R = arginine, S/T = phospho-S/T, Q = glutamine, G = glycine, K = lysine, P = proline, X = any amino acid.

**Fig. 6:**
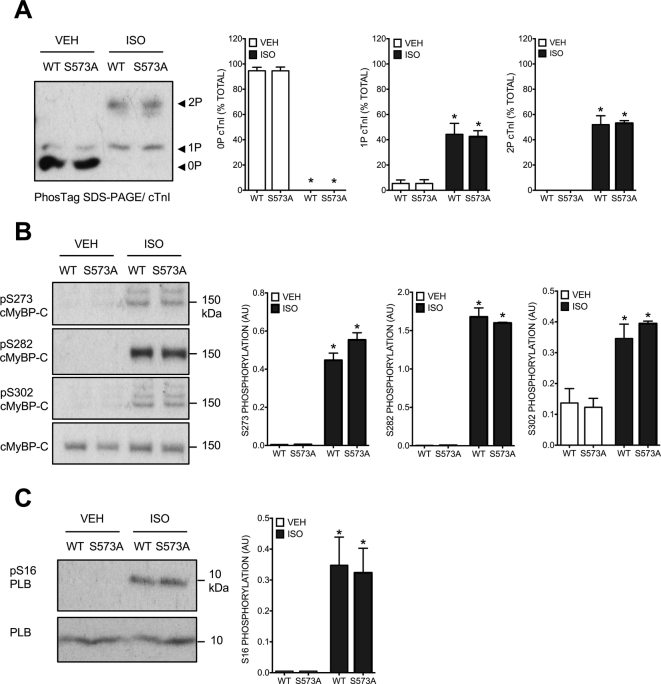
Phosphorylation of specific cardiac phosphoproteins in transduced ARVM. ARVM transduced with AdV.GFP-B56δ-WT or AdV.GFP-B56δ-S573A were maintained in culture for 18 h and exposed to vehicle (VEH) or isoprenaline (ISO). A. Representative immunoblot and quantitative data (mean ± SEM) show abundance of non-phosphorylated (0P), mono-phosphorylated (1P) and bis-phosphorylated (2P) cTnI. 0P, 1P and 2P cTnI are expressed as a percentage of total cTnI, which is the sum of the signals in each lane. *P < 0.05 vs corresponding VEH (*n* = 3). B. Representative immunoblots and quantitative data (mean ± SEM) show phosphorylation of cMyBP-C at S273, S282 and S302. *P < 0.05 vs corresponding VEH (*n* = 3). C. Representative immunoblots and quantitative data (mean ± SEM) show phosphorylation of PLB at S16. *P < 0.05 vs corresponding VEH (*n* = 3).

To explore the potential role of B56δ phosphorylation at S573 in β-adrenergic regulation of cardiac protein phosphorylation, we performed immunoblot analyses of the ARVM phosphoproteome using a selection of phospho-specific antibodies detecting phospho-S/T residues in distinct kinase substrate motifs (Fig. 5). Only a limited number of ISO-responsive phosphoproteins were detected by these antibodies, and the intensity of those signals did not differ between cells expressing GFP-B56δ-WT and those expressing GFP-B56δ-S573A. Of note, the phospho-specific antibody recognizing the RRXS/T motif detected phosphorylation of GFP-B56δ-WT but not GFP-B56δ-S573A (Supplementary Fig. 5), confirming the ability of the method to reveal differential phosphorylation of S/T residues in abundantly expressed proteins. In complementary experiments, we determined the phosphorylation status of cTnI, cardiac myosin binding protein-C (cMyBP-C) and phospholamban (PLB), which are phosphorylated by PKA following βAR stimulation and play important roles in mediating inotropic and lusitropic responses. PhosTag SDS/PAGE-immunoblot analysis of cTnI showed that the abundance of non-phosphorylated (0P), mono-phosphorylated (1P) and bis-phosphorylated (2P) cTnI was comparable in cells expressing GFP-B56δ-WT and those expressing GFP-B56δ-S573A, in the absence or presence of ISO stimulation (Fig. 6A). Basal and ISO-induced phosphorylation of cMyBP-C at S273, S282 and S302 (Fig. 6B), and PLB at S16 (Fig. 6C) was also comparable between cells expressing GFP-B56δ-WT and those expressing GFP-B56δ-S573A.

In complementary experiments, we determined the relative contribution of PP2A versus PP1 activity to the dephosphorylation of cTnI, cMyBP-C and PLB, by assessing the impact of treatment with OA at a concentration (100 nM) that selectively inhibits PP2A [38] or at a higher concentration (1 μM) that also inhibits PP1 [39]. When used at a PP2A-selective concentration, OA increased the phosphorylation of both cTnI at S22/23 and cMyBP-C at S282 (Supplementary Fig. 6). In contrast, PLB phosphorylation at S16 was increased only when PP1 was also inhibited (Supplementary Fig. 6). The responses to treatment with different concentrations of OA were comparable in ARVM expressing GFP-B56δ-WT or GFP-B56δ-S573A (Supplementary Fig. 6). These findings suggest that PP2A activity is primarily responsible for dephosphorylating cTnI at S22/23 and cMyBP-C at S282, with PP1 activity primarily responsible for dephosphorylating PLB at S16, and additionally indicate that those contributions of PP2A versus PP1 activity are not modified by ablation of S573 in heterologously expressed GFP- B56δ.

### 3.6 Expression and phosphorylation of B56δ in mouse models of cardiac hypertrophy

**Fig. 7:**
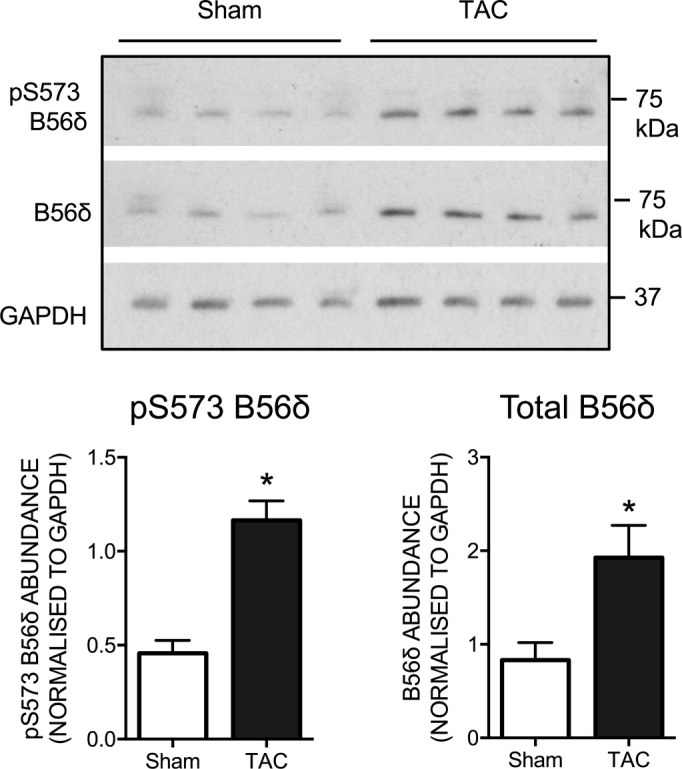
B56δ regulation in mouse cardiac hypertrophy. Immunoblots and quantitative data (mean ± SEM) show the abundance of phosphorylated (pS573) and total B56δ in mouse hearts 7 days after TAC or sham surgery. *P < 0.05 vs sham. (*n* = 4).

Finally, to explore whether the phosphorylation and/or the expression of cardiac B56δ is altered in a pathophysiological setting, we examined these in a mouse model of pressure overload-induced cardiac hypertrophy. Seven days post-surgery, heart weight/tibia length was 10.1 ± 0.8 mg/mm in mice subjected to TAC and 7.7 ± 0.2 mg/mm in mice subjected to sham operation (n = 7, P < 0.05), reflecting cardiac hypertrophy in the former. The abundance of both pS573 B56δ and total B56δ was increased in the hearts of mice subjected to TAC relative to mice subjected to sham operation (Fig. 7). These findings suggest that, while the relative phosphorylation of B56δ is unaltered, the absolute abundance of pS573 B56δ is increased in the setting of pressure overload-induced cardiac hypertrophy.

## 4 Discussion

The present study has revealed for the first time in a cardiac context that (i) acute simulation of βARs induces a rapid and sustained phosphorylation of the PP2A regulatory subunit B56δ selectively at S573, (ii) this phosphorylation occurs principally through stimulation of the β_1_AR and activation of PKA, and (iii) βAR/PKA-mediated phosphorylation of B56δ at S573 increases PP2A activity.

In healthy myocardium, catecholamines regulate a variety of cellular processes primarily by β_1_AR-mediated activation of protein kinases [40], in particular the Ser/Thr kinases PKA and calcium/calmodulin-dependent kinase II (CaMKII), and the downstream phosphorylation of PKA and CaMKII substrate proteins [21], [22], [41], [42]. Although the regulation and physiological roles of PKA and CaMKII have been studied extensively [43], [44], [45], knowledge of potential β_1_AR-mediated regulation of Ser/Thr phosphatases that serve an opposing role is restricted largely to type 1 phosphatase (PP1), whose activity is regulated through PKA-mediated phosphorylation of the PP1-specific inhibitor of protein phosphatase-1 (I-1) [46], [47]. Nevertheless, members of the PP2A family are also believed to contribute significantly to myocardial Ser/Thr phosphatase activity and thus dephosphorylation of functionally important Ser/Thr residues in cardiomyocyte proteins [30], [48], [49], [50], [51]. Furthermore, there is emerging evidence for βAR-mediated regulation of PP2A through it regulatory B subunits, for example through translocation of the B56α isoform between subcellular compartments [29].

Here, we provide robust new evidence that in ARVM the non-selective βAR agonist ISO induces phosphorylation of the PP2A regulatory subunit isoform B56δ selectively at S573, in a rapid and sustained manner (Fig. 1). Our data with CGP (a selective β_1_AR antagonist) and ICI (a selective β_2_AR antagonist) indicate that the ISO response is mediated primarily by the β_1_AR subtype (Fig. 2A). In cells that express each receptor at similar abundance, the affinity of CGP for the β_1_AR is comparable to that of ICI for the β_2_AR [52]. Importantly, in our experiments, ISO-induced phosphorylation of cTnI at S23/24 (which in ARVM occurs through the β_1_AR) [53], [54] was also abolished by CGP but not ICI at a concentration of 100 nM for each agent, indicating effective blockade of β_1_AR signaling by the former antagonist. On the basis of this observation and evidence that in ARVM the relative β_1_AR:β_2_AR abundance is 65:35 [55], it is reasonable to assume that 100 nM ICI was sufficient to block β_2_AR activation and that its relative ineffectiveness in inhibiting ISO-induced B56δ phosphorylation arose from a minor role for this βAR subtype in mediating that response.

The data that we have obtained from complementary studies, in which we explored the role of PKA in mediating B56δ phosphorylation at S573, indicate that activation of this kinase is both necessary for the ISO-induced response and sufficient per se to induce such phosphorylation. Thus, ISO-induced B56δ phosphorylation was significantly attenuated by pre-treatment of ARVM with either H89 or PKI, which inhibit PKA through distinct mechanisms [56], and selective PKA activation by the cAMP analog BNZ induced B56δ phosphorylation that was similar in magnitude to that induced by ISO (Fig. 2B and C).

We have shown previously that βAR stimulation by ISO in ARVM alters the subcellular localisation of another PP2A regulatory subunit isoform, B56α [29]. Furthermore, in non-cardiac cells, phosphorylation of B56α has been reported to induce a change in subcellular localisation of the B56α-PP2A holoenzyme [57]. These findings prompted us to explore the possibility that B56δ phosphorylation might impact on the subcellular localisation of this PP2A regulatory subunit in ARVM. Nevertheless, there was no marked response to ISO in experiments utilizing immunolabeling and fluorescent confocal microscopy (which revealed the spatial distribution of B56δ in intact cells; Fig. 3A) or cellular fractionation and immunoblot analysis (which revealed the relative abundance of B56δ in soluble and insoluble fractions; Fig. 3B), indicating that phosphorylation at S573 does not regulate the subcellular localization of B56δ in ARVM. Furthermore, B56δ was found to be present throughout the cytosol and nuclei of ARVM (Fig. 3A), which is consistent with the B56δ distribution reported in rat brain cells [58] and neuronally differentiated PC12 cells [19], suggesting that B56δ-PP2A might target phosphoproteins in multiple subcellular compartments.

To confirm if S573 is the only B56δ residue that is phosphorylated downstream of βAR stimulation in a cellular context and to explore potential functional roles of such phosphorylation, we transduced ARVM with adenoviruses expressing GFP-tagged B56δ in WT or S573A mutant form. This work confirmed that, following βAR stimulation, S573 is the only B56δ residue that exhibits increased phosphorylation, as detected by a phospho-S/T antibody that targets the optimal PKA substrate motif (Fig. 4C and Supplementary Fig. 4B). An interesting observation that arose from the analysis of transduced ARVM was the approximately 5-fold increase in the expression of endogenous PP2A_C_ protein that accompanied the heterologous expression of GFP-tagged B56δ in either form (Fig. 4A). Of note, upregulated PP2A_C_ expression in response to increased expression of a B56 isoform is not unique to our present work in isolated ARVM and has been reported previously in hearts of transgenic mice with cardiac-specific overexpression of B56α [10]. Interestingly, expression of endogenous PP2A_A_ protein was unchanged following heterologous expression of GFP-B56δ-WT or GFP-B56δ-S573A (Fig. 4A). Thus it appears that in ARVM PP2A_C_ expression is stringently regulated and increases only when additional regulatory subunits are available, but there may be an excess pool of PP2A_A_.

We also explored whether the heterologously expressed of GFP-tagged B56δ variants formed functional PP2A holoenzyme complexes with endogenous PP2A_C_ and PP2A_A_ proteins. Analysis of GFP immunocomplexes from ARVM expressing GFP-B56δ-WT or GFP-B56δ-S573A (but not those expressing GFP alone) revealed the presence of PP2A_A_ and PP2A_C_ proteins in comparable abundance (Fig. 4B), suggesting that the GFP-tagged B56δ variants formed heterotrimeric PP2A holoenzymes with endogenous scaffolding and catalytic subunits. Furthermore, following ISO stimulation, PP2A activity (likely arising largely from newly formed holoenzyme complexes incorporating the upregulated PP2A_C_ subunits) was increased in ARVM expressing GFP-B56δ-WT but not those expressing GFP-B56δ-S573A (Fig. 4E), indicating that S573 phosphorylation in B56δ is essential for a βAR-mediated increase in the activity of associated catalytic subunits. This is consistent with previous observations in non-cardiac cells with different stimuli that also increase cellular PKA activity [16].

Finally, we investigated whether ISO-induced phosphorylation of cardiomyocyte proteins differed between ARVM expressing GFP-B56δ-WT versus GFP-B56δ-S573A. Somewhat surprisingly, phospho-specific antibodies targeted at phospho-S/T residues residing within distinct kinase substrate motifs detected only a limited number of phosphoproteins that displayed altered phosphorylation in response to ISO stimulation, with little apparent difference between cells expressing GFP-B56δ-WT or GFP-B56δ-S573A (Fig. 5). The most abundant ISO-responsive phosphoproteins that were detected migrated at molecular weights that correspond to those of cTnI, cMyBP-C and PLB, which are known PKA targets that are phosphorylated in response to βAR stimulation in cardiomyocytes [23], [42], [59]. Thus, we explored the relative abundance of non-phosphorylated (0P), mono-phosphorylated (1P) and bis-phosphorylated (2P) cTnI moieties, and determined the phosphorylation of cMyBP-C and PLB at known phosphoacceptor residues of established functional importance (S273, S282 and S302 in cMyBP-C; S16 in PLB). However, in the absence or presence of ISO stimulation, the phosphorylation profiles of cTnI, cMyBP-C and PLB were not different between ARVM expressing GFP-B56δ-WT and those expressing GFP-B56δ-S573A (Fig. 6). Although these observations may suggest that the phosphorylation status of S573 in B56δ has no influence on βAR-mediated phosphorylation of functionally important phosphoproteins, including cTnI, cMyBP-C and PLB, some observations temper such a conclusion. Firstly, while the heterologously expressed GFP-B56δ-WT and GFP-B56δ-S573A proteins appeared to form new and functional PP2A holoenzymes whose activity was regulated in a manner that depended on B56δ phosphorylation at S573, these might not have displaced pre-existing endogenous holoenzymes that are co-localized with their substrates. Indeed, in ARVM expressing GFP-B56δ-WT or GFP-B56δ-S573A the abundance and ISO-induced phosphorylation of endogenous B56δ were unaltered (Fig. 4C). Secondly, since the phosphospecific antibodies used detected only a subset of abundantly expressed phosphoproteins (Fig. 5), our data do not preclude the existence of other phosphoproteins that are not detected by this approach but are nevertheless regulated by B56δ-PP2A upon βAR stimulation, in a manner that is dependent on B56δ phosphorylation at S573. Notably, it has been proposed that PKA and B56δ-PP2A are co-localized by the anchoring protein mAKAP, to form a signaling complex that regulates the phosphorylation and activity of the phosphodiesterase isoform PDE4D3 and thereby the local concentration of cAMP [60]. Nevertheless, the pertinent evidence was obtained from studies with heterologous protein expression in HEK 293 cells, and the physiological significance of this signaling pathway in cardiomyocytes remains to be confirmed.

With regard to the potential (patho)physiological role(s) of B56δ phosphorylation, our data from an exploratory analysis of mouse hearts with pressure overload-induced cardiac hypertrophy show that, while the relative phosphorylation of B56δ was unaltered, the absolute abundance of pS573 B56δ was increased significantly in that setting. This change may be a compensatory response to pressure overload-induced cardiac hypertrophy, or play a mechanistic role in its pathogenesis. Notwithstanding, the dynamic regulation of B56δ phosphorylation and expression in cardiac hypertrophy warrants investigation of the functional consequences of their targeted manipulation in appropriate models. In that context, it is interesting to note also that B56δ expression has been reported to be increased in dog hearts following myocardial infarction and in the setting of tachypacing-induced non-ischemic heart failure [20], although B56δ phosphorylation status was not determined in that study.

In conclusion, the present study has identified and characterised a novel mechanism that is likely to regulate a specific pool of PP2A activity in cardiomyocytes in response to βAR stimulation, through PKA-mediated phosphorylation of the PP2A regulatory subunit isoform B56δ at S573. The functional importance of this novel mechanism now requires the establishment of a model system that allows replacement of endogenous B56δ with a non-phosphorylatable (S573A) mutant (e.g. a knock-in mouse) and the application of broad-spectrum phosphoproteomics analyses, as well the examination of cardiac responses to (patho)physiological stimuli, in such a model.

## Disclosures

None.

## Appendix A Supplementary data

Supplementary material Image 1
