# Supplementary material for: β-Adrenergic regulation of cardiac type 2A protein phosphatase through phosphorylation of regulatory subunit B56δ at S573
Source: J Mol Cell Cardiol. 2018 Feb;115:20–31. doi: 10.1016/j.yjmcc.2017.12.016 (PMC5823843; doi:10.1016/j.yjmcc.2017.12.016)
Supplement: Supplementary file 1 — Supplementary material [file mmc1.pdf]

β-Adrenergic regulation of cardiac type 2A protein phosphatase through phosphorylation of regulatory subunit B56δ at S573

Antonella Ranieri, PhD; Elizabeth Kemp, PhD; Joseph R Burgoyne, PhD; Metin Avkiran, PhD, DSc

School of Cardiovascular Medicine and Sciences, King's College London British Heart Foundation Centre of Research Excellence, The Rayne Institute, St Thomas' Hospital, London, United Kingdom

Supplementary Figures 1-6

Supplementary Figure 1

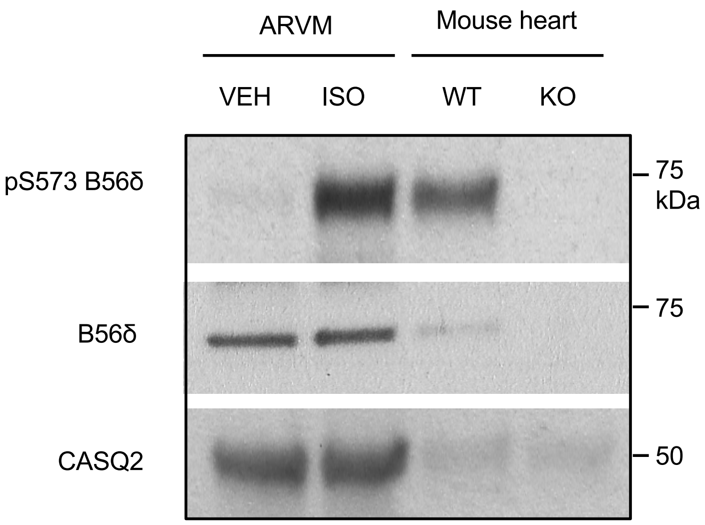

Validation of the B56δ total and phospho-S573 antibody. ARVM exposed to vehicle (VEH) or isoprenaline (ISO), and cardiac tissue of littermate WT and B56δ KO mice were subjected to immunoblot analysis with the B56δ phospho-S573 and total antibody. Confirming the specificity of both antibodies, the protein detected at ~75-kDa in ARVM and in WT mouse cardiac tissue was not detected in B56δ KO cardiac tissue.

Supplementary Figure 2

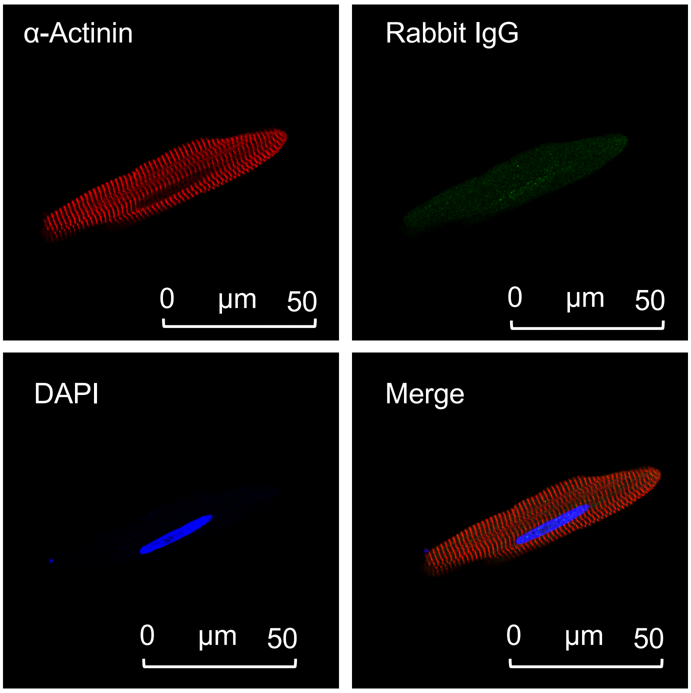

Non-immune rabbit IgG Cy5 fluorescence. Fixed and permeabilized (unstimulated) ARVM were incubated first with α-actinin primary antibody and non-immune rabbit IgG, then with Cy3-anti-mouse and Cy5-anti-rabbit secondary antibodies. Nuclei were stained with DAPI. Representative images show Cy3-labeled α-actinin (red), Cy5-labelled rabbit IgG (green) and DAPI-stained nuclei (blue) in separate channels. A Merged image is also shown.

Supplementary Figure 3

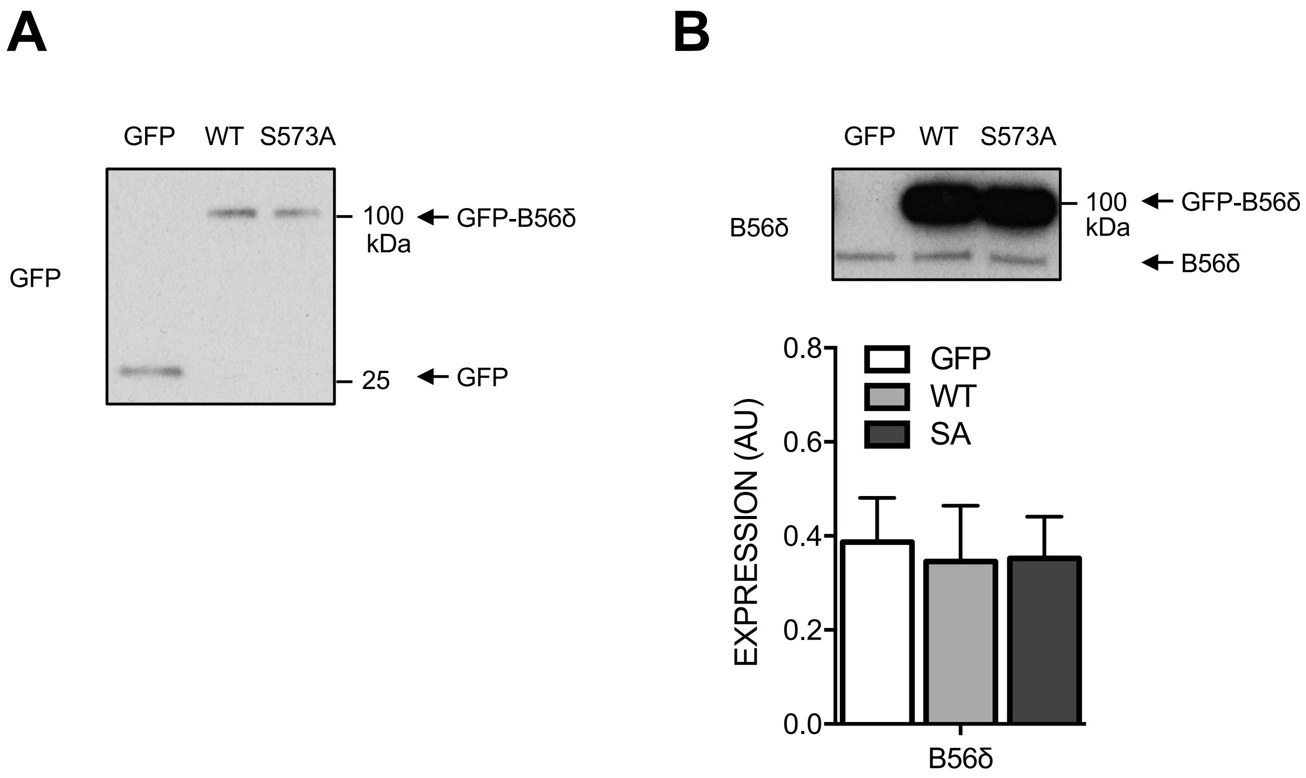

GFP, GFP-B56δ-WT and GFP-B56δ-S573A expression in ARVM. ARVM transduced with AdV.GFP (MOI 30), AdV.GFP-B56δ-WT or AdV.GFP-B56δ-S573A (MOI 100) were maintained in culture for 18 hours. **A.** Expression of GFP, GFP-B56δ-WT and GFP-B56δ-S573A, as determined by immunonblot analysis with the GFP antibody. **B.** Expression of GFP-B56δ-WT, GFP-B56δ-S573A and endogenous B56δ, as determined by immunonblot analysis with the B56δ antibody. Quantitative data (mean ± SEM) show expression of endogenous B56δ in transduced cells (*n*=3).

Supplementary Figure 4

A

```
MPYKLKKEKE PPKVAKCTAK PSSSGKDGGG ENTEEAQPQP QPQPQPQAQS 50
QPPSSNKRPS NSTPPPTQLS KIKYSGGPQI VKKERRQSSS RFNLSKNREL 100
QKLPALKDSP TQEREELFIQ KLRQCCVLFD FVSDPLSDLK FKEVKRAGLN 150
EMVEYITHSR DVVTEAIYPE AVTMFSVNLF RTLPPSSNPT GAEFDPEEDE 200
PTLEAAWPHL QLVYEFFLRF LESPDFQPNI AKKYIDQKFV LALLDLFDSE 250
DPRERDFLKT ILHRIYGKFL GLRAYIRRQI NHIFYRFIYE TEHHNGIAEL 300
LEILGSIING FALPLKEEHK MFLIRVLLPL HKVKSLSVYH PQLAYCVVQF 250
LEKESSLTEP VIVGLLKFWP KTHSPKEVMF LNELEEILDV IEPSEFSKVM 400
EPLFRQLAKC VSSPHFQVAE RALYYWNNEY IMSLISDNAA RVLPIMFPAL 450
YRNSKSHWNK TIHGLIYNAL KLFMEMNQKL FDDCTQQYKA EKQKGRFRMK 500
EREEMWQKIE ELARLNPQYP MFRAPPPLPP VYSMETETPT AEDIQLLKRT 550
VETEAVQMLK DIKKEKVLLR RKSELPQDVY TIKALEAHKR AEEFLTASQE 600
AL
```

B

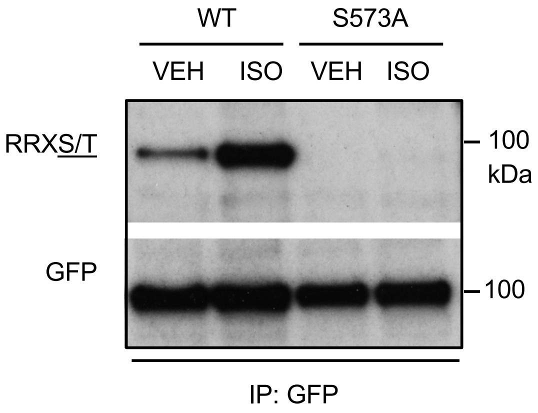

Phosphorylatable serine residues in human B56δ. A. Amino acid sequence of human B56δ. Motifs of interest are in bold. S88 (indicated by a single line) and S573 (indicated by a double line) are preceded by arginine at -2 and -3 positions. A phospho-specific antibody detecting phospho-S/T residues in the RRX<u>S/T</u> motif would detect both phospho-S88 and phospho-S573. **B.** ARVM transduced with AdV.GFP-B56δ-WT or AdV.GFP-B56δ-S573A (MOI 100) were maintained in culture for 18 hours and exposed to vehicle (VEH) or isoprenaline (ISO). Immunoprecipitated GFP-B56δ-WT and GFP-B56δ-S573A were subjected to immunoblot analysis with a phospho-specific antibody recognizing phospho-S/T residues in the RRX<u>S/T</u> motif. R=arginine, <u>S/T</u>=phospho-S/T, X=any amino acid.

Supplementary Figure 5

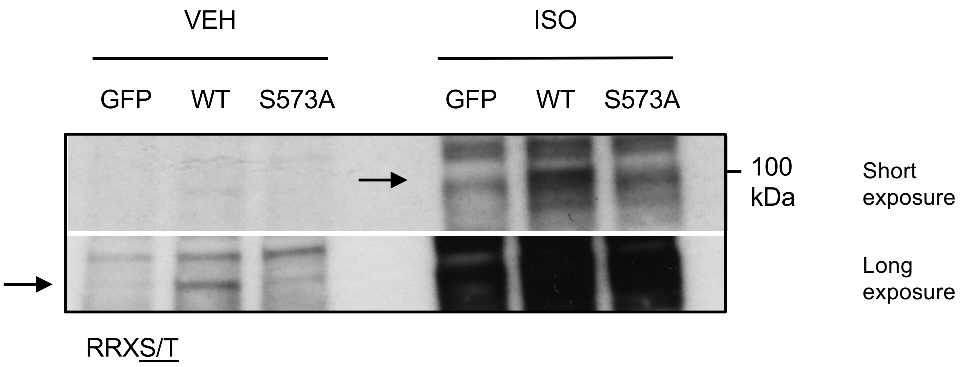

Basal and ISO-induced phosphorylation of GFP-56δ-WT. ARVM transduced with AdV.GFP-B56δ-WT or AdV.GFP-B56δ-S573A (MOI 100) were maintained in culture for 18 hours and exposed to vehicle (VEH) or isoprenaline (ISO). Immunoblot analysis of the ARVM phosphoproteome was performed with a phospho-specific antibody detecting phosphorylated S/T residues in the RRXS/T motif. Short and long exposures of the film to the ECL signal are shown. Arrows indicate basal and ISO-induced phosphorylation of heterologous B56δ in cells expressing GFP-B56δ-WT. R=arginine, S/T=phospho-S/T, X=any amino acid.

Supplementary Figure 6

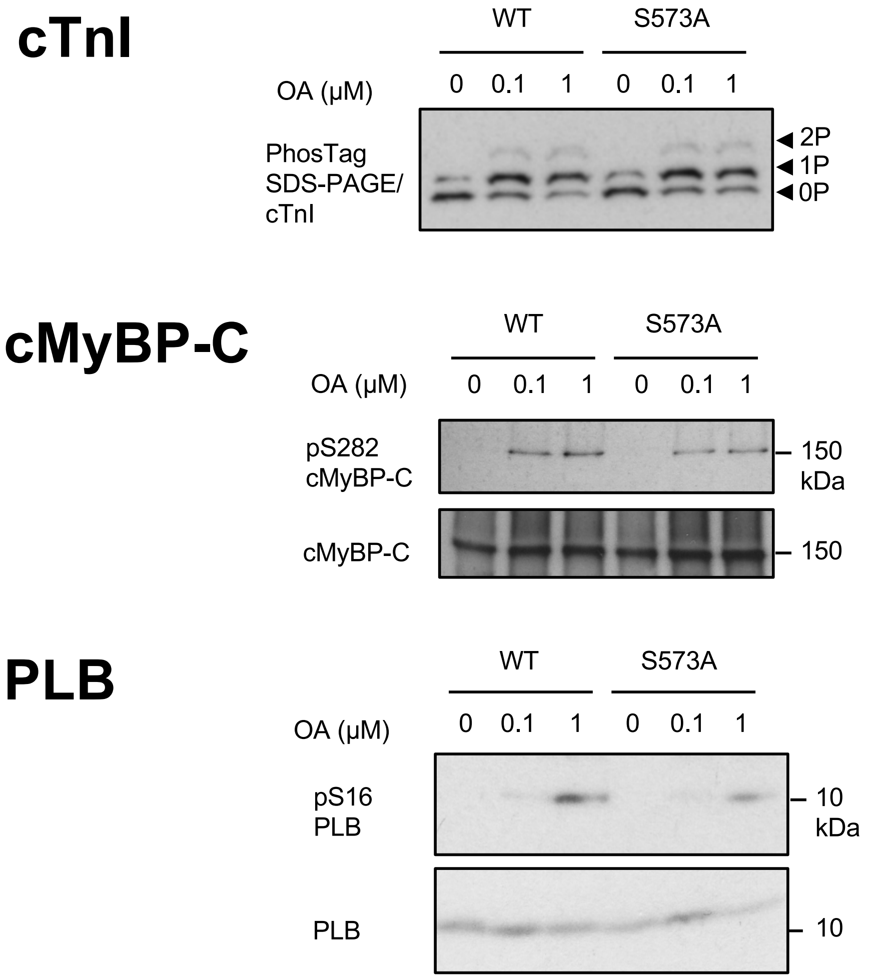

Cardiac protein phosphorylation in the presence of okadaic acid. ARVM transduced with AdV.GFP-B56δ-WT or AdV.GFP-B56δ-S573A (MOI 100) were maintained in culture for 18 hours. Cells were exposed to vehicle, 0.1 or 1 μM okadaic acid (OA). Immunoblots show abundance of non-phosphorylated (0P), mono-phosphorylated (1P) and bis-phosphorylated (2P) cTnI, phosphorylation of cMyBP-C at S282 and phosphorylation of PLB at S16.
